# Burden of Illness in Follicular Lymphoma with Multiple Lines of Treatment, Italian RWE Analysis

**DOI:** 10.3390/cancers15174403

**Published:** 2023-09-02

**Authors:** Andrés J. M. Ferreri, Pier Luigi Zinzani, Carlo Messina, Diletta Valsecchi, Maria Chiara Rendace, Eleonora Premoli, Elisa Giacomini, Chiara Veronesi, Luca Degli Esposti, Paola Di Matteo

**Affiliations:** 1 Unità Linfomi, IRCCS Ospedale San Raffaele, 20132 Milan, Italy; ferreri.andres@hsr.it; 2 Istituto di Ematologia “Seràgnoli”, IRCCS Azienda Ospedaliero-Universitaria di Bologna, 40138 Bologna, Italy; pierluigi.zinzani@unibo.it; 3 Dipartimento di Scienze Mediche e Chirurgiche, Università di Bologna, 40126 Bologna, Italy; 4 Novartis Farma S.p.A., 20154 Milan, Italy; messina.carlo@me.com (C.M.); diletta.valsecchi@novartis.com (D.V.); maria_chiara.rendace@novartis.com (M.C.R.); eleonora_premoli@hotmail.com (E.P.); paola.di_matteo@novartis.com (P.D.M.); 5 CliCon S.r.l. Società Benefit, Health, Economics & Outcomes Research, Via Murri, 40137 Bologna, Italy; elisa.giacomini@clicon.it (E.G.); chiara.veronesi@clicon.it (C.V.)

**Keywords:** chemotherapy, follicular lymphoma, indolent B-cell lymphoma, non-Hodgkin lymphoma, real world evidence, treatment lines

## Abstract

**Simple Summary:**

This study analysed the state-of-the-art of follicular lymphoma in Italy in a real-world clinical setting, giving special attention to patients who underwent three or more treatment lines. The overall message emerging from the analysis is that progression through several lines during the management of patients diagnosed with follicular lymphoma results in more complex clinical status, and more relevant financial implications for sustainability by the National Health Service. Thus, at the forefront of efforts of current research to broaden the landscape of therapeutic strategies to manage this disease, the mortality rates are still high, revealing an unmet clinical need for patients with multiple lines.

**Abstract:**

This real-world analysis investigated patients with follicular lymphoma in Italy receiving three or more treatment lines (≥3L), focusing on therapeutic pathways with their rebounds on healthcare resource consumptions and costs. Data were retrieved from administrative databases from healthcare entities covering about 13.3 million residents. Adults diagnosed with follicular lymphoma were identified between January 2015 and June 2020, and among them 2434 patients with ≥3L of treatment during the data availability interval (January 2009 to June 2021) were included. Of them, 1318 were in 3L, 494 in 4L and 622 in ≥5L. A relevant proportion of patients (12–32%) switched to a later line within the same calendar year. At 3-year follow-up (median), 34% patients died. Total mean annual expenses were euro 14,508 in the year preceding inclusion and rose to euro 21,081 at 1-year follow-up (on average euro 22,230/patient/year for the whole follow-up), with hospitalization and drug expenses as weightiest cost items. In conclusion, the clinical and economic burden of follicular lymphoma increases along with later treatment lines. The high mortality rates indicate that further efforts are needed to optimize disease management.

## 1. Introduction

Follicular lymphoma is an indolent B-cell lymphoma, being the second most common form among all non-Hodgkin lymphomas (NHLs) [1,2]. Like other Western countries [3], data on the Italian population showed an average onset age between 60 and 65 years, a comparable distribution between males and females, and a relative frequency among all NHL subtypes of 18.55% [4].

Up to about twenty years ago, the median overall survival (OS) associated to follicular lymphoma was below 10 years, but the growing availability of novel treatment options has dramatically changed the life expectancy of these patients, up to 20 years in most cases [5]. This impressive progress has been possible following the introduction of anti-CD20 antibodies that provide significant benefits as first line therapy, as well as in subsequent lines for patients with disease progression [6].

This disease is commonly characterized by a remitting relapsing course and the initial management ranges from the so-called “watch-and-wait” (active monitoring/observation) approach to immediate frontline treatment with chemotherapy (CT), radiotherapy, or rituximab induction therapy [7].

The most recent guidelines of the European Society for Medical Oncology (ESMO) recommend a treatment approach based on the patient’s characteristics and extension of disease [8]. In brief, ESMO advises a watch-and-wait strategy for patients with asymptomatic, low tumour burden disease, radiation therapy with curative intent for the less common cases of limited-stage disease, and systemic chemoimmunotherapy for those with disseminated disease who achieve GELF criteria [8]. Despite several therapeutic improvements, follicular lymphoma tends to disseminate and affected patients experience multiple relapses, progressively with a shorter interval between failures, and with less responsive and more aggressive disease after each recurrence.

Patients who experience relapsed or refractory disease need to proceed beyond more lines of therapy [9,10]. The management of these patients has evolved over time thanks to the introduction of new classes of agents, such as phosphoinositide 3-kinase inhibitors (idelalisib) [11], epigenetic therapies [12], immunomodulators (lenalidomide) [13,14], and chimeric antigen receptor T cells [15]. In the last few years, different combinations of novel molecules with approaches already in use, based on chemotherapy (CT), anti-CD20 antibodies (i.e., rituximab, obinutuzumab, ibritumomab tiuxetan), radioimmunotherapy, radiation therapy, and autologous stem cell transplantation (ASCT) [6] have received approval by drug regulatory authorities [16], including the Italian Medicine Agency (AIFA), as therapy for previously treated patients with follicular lymphoma [17,18,19].

Nonetheless, the setting of relapsed/refractory follicular lymphoma is a still open clinical challenge, given the multiple factors affecting therapeutic choices, such as the number and type of prior treatment lines, response duration, disease burden at relapse, and the patient’s individual clinical conditions.

There is large evidence to show that the clinical and economic burden of follicular lymphoma is high and management costs increase along with the progression of both the disease itself and the sequential lines of treatment [20,21,22].

The present analysis, conducted in a real-world Italian clinical setting, was aimed at evaluating the number of patients affected by follicular lymphoma receiving three or more lines of treatment, characterizing the demographic and clinical characteristics of this population stratified by treatment line and year of inclusion, analysing the therapeutic pathways and drug utilization (for lines of therapy), and assessing the consumption of healthcare resources with the related direct costs sustained by the Italian National Health Service (INHS).

## 2. Materials and Methods

### 2.1. Data Source

In Italy, healthcare is provided to all citizens and residents by a mixed public–private system. The public part is the INHS, which is administered on a regional basis (20 regions). Each region is divided into Local Health Units (LHUs), which are administrative bodies with the function of delivering health services. LHUs manage the administrative databases. Such databases hold information that is meant to be used for administrative purposes in order to track the economic flows from the INHS to the healthcare providers for reimbursement purposes. Administrative databases allow the identification and description of medicine use profiles in daily clinical practice. Within administrative databases, information is recorded according to an anonymous patient ID code and the date of the service provision, in order to meet the different needs of the administration. This system allows us to collect and link together all information available for each subject, in respect of privacy laws, and to draw an analytical and chronological profile of services supplied. In view of the principle of universal healthcare coverage through INHS, these databases are repositories of data meant for reimbursement purposes, warranted to all citizens and foreign residents. For the present analysis, data were retrieved from the administrative databases of a pool of healthcare providers geographically distributed throughout Italy, covering about 13.3 million health-assisted residents. As previously described by our group [23], the administrative databases browsed for the analysis were the following: demographic database (for age, sex, and death information), pharmaceutical database (to track data related on the drug prescription dispensed), hospitalization database (to get hospitalization-related data such as diagnosis, hospitalization date, and cost), outpatient specialist services (OSS) database (to collect data on specialistic visits or test prescription).

In order to guarantee privacy, in full compliance with the European General Data Protection Regulation (GDPR) (2016/679), study participants were identified through an anonymous univocal numeric code, that allowed the electronic linkage between the various databases. All the results of this analysis were provided in aggregated form, so that any information could not be attributable, either directly or indirectly, to individual patients.

### 2.2. Identification of Study Population and Study Periods

From January 2015 to June 2020 (inclusion period), adult patients with a hospital discharge diagnosis for follicular lymphoma, identified by International Classification of Diseases, Ninth Revision, Clinical Modification (ICD-9-CM) code 202.0 within the hospitalization database, were selected. The lines of treatment were analysed considering all data available (from January 2009 to June 2021), and only the patients with at least 3 treatment lines (≥3L) were included. The third line was not necessarily administered during the inclusion period. The population was stratified according to the number of treatment lines as follows: (i) patients in the third line (3L); (ii) patients in the fourth line (4L); and (iii) patients in the fifth line or subsequent lines (≥5L). The index date was the time of the start of the third treatment line. Patient’s clinical history was characterized during a 12-month pre-index period. The follow-up was the timespan from the index date until the end of the data availability period or date of death (whatever occurred first).

### 2.3. Demographic and Clinical Characteristics of the Study Population

For all the patients, age at index date and gender were recorded. The clinical characteristics were assessed during the pre-index period evaluating the comorbidity profile through the Charlson comorbidity index (CCI), a scoring system that results from the sum of a weight assigned to each concomitant disease [24]. Since these diseases were proxied by searching drug prescriptions (through the Anatomical Therapeutic Chemical-ATC codes in the pharmaceutical databases) and hospitalization discharge codes (according to ICD-9-CM), untreated and non-hospitalized comorbidities could not be captured. In this specific analysis, a modified version of the CCI, not accounting for cancer, was applied. To better characterize the patients’ clinical pictures, the most frequently dispensed medications and the most common all-cause hospitalizations were also recorded in the year before the index date and during the entire available follow-up.

### 2.4. Patterns and Definition of Treatment Lines

The list of treatments and procedures with the related codes included in the present analysis to evaluate the pattern of treatment lines are provided in Supplementary Table S1. For the included population of patients with follicular lymphoma, the number of treatment lines were determined by looking at the prescription for drugs and procedures (including autologous stem cell transplantation—ASCT) among those found during the whole period of data availability. Within administrative databases, chemotherapy is frequently tracked by a chemotherapy (CT) infusion procedure without identification of the specific drug infused, due to the low cost of some drugs used in CT regimens. Therefore, it is not always possible to correctly identify a change of line between two consecutive chemotherapeutic records, and a proxy was applied in order to estimate the number of lines between consecutive CTs. Specifically, a gap of 4 months between consecutive aspecific CT records or 6 months between consecutive rituximab plus CT records was considered as a progression to a successive line, based on the assumption that 4–6 cycles are usually administered [8]. No time gap was applied for the other therapies/procedures or in case of rituximab as monotherapy.

Subjects who had received only aspecific CT and were then diagnosed with follicular lymphoma afterwards, and those who had a diagnosis of other cancer prior to aspecific CT, were considered without treatment in the analysis.

Patients who underwent at least 3 lines of treatment were then analysed for the distribution across the various treatment lines with the related follow-up length (expressed as median and mean ± SD), calculated starting from the beginning of each treatment line. The included patients were also analysed for each complete year of the inclusion period (2015 to 2020) and stratified by number of treatment lines. The following variables were collected for every inclusion year (2020 not reported as inclusion ended at June): prevalent patients, as in patients that, up to the specific calendar year, were in a certain line number (as in currently treated or treated in the previous year); incident to line (patients who started the line within the calendar year); switch to subsequent line (patients moving towards lines of therapy within each calendar year); deceased patients. The incident patients observed within each calendar year were then re-proportioned to the Italian population.

### 2.5. Healthcare Resource Consumptions and Costs

The healthcare resource use and related costs for the INHS in patients with at least three lines of treatment were examined during the characterization period and follow-up. The analysis of healthcare resource use considered the annual number per patient (expressed as mean ± standard deviation) of drug treatments, specialistic visits, diagnostic services, and hospitalizations. The resulting direct healthcare costs per patient in euros, by item and overall, were evaluated during the year before index date (characterization period), during the first and second years, and for the whole available follow-up.

### 2.6. Statistical Analysis

Descriptive statistics were carried out for continuous variables presented as mean ± standard deviation (SD) or medians, and categorical variables given as numbers and percentages. Kaplan Meier curves were used to analyse overall survival of patients with follicular lymphoma with ≥3 lines of treatment. Survival was calculated as the time (in months) from therapy initiation to the date of death for any cause plus one day. For alive patients, overall survival was censored at the date of database availability (censoring rule). All analyses were carried out using STATA SE, version 17.0 (StataCorp LLC, College Station, TX, USA).

## 3. Results

Among the study sample covering a catchment area of about 13.3 million residents, 8406 diagnoses of follicular lymphoma were found starting from January 2015. Among them, 7021 patients had a hospital discharge diagnosis for follicular lymphoma with at least one year of data availability before and after hospitalization. Of these, 5650 (80.5%) were receiving a treatment or procedure during all the study period, and 2434 had at least three lines of treatment. The total 2434 patients with ≥three lines had a median follow-up duration of 2.9 years. Of them, 1318 were in 3L (median follow-up 2.1 years), 494 in 4L (median follow-up 2.1 years), 622 in ≥5L (median follow-up 3.1 years).

**Figure 1:**
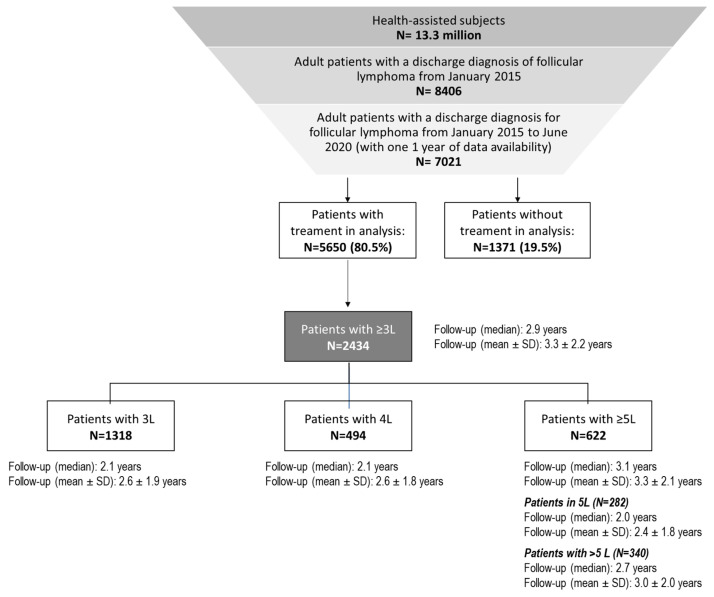
Flowchart for the selection of patients diagnosed with follicular lymphoma with at least 3 lines of treatment, and distribution of the 2434 patients with ≥3L across the different lines (from the third onward). The follow-up length (years) in each subgroup has been calculated starting from the beginning of the related treatment line and expressed as median and mean ± SD.

The flowchart of patients’ selection and the number of patients in each line of treatment from the third onward with the follow-up duration in each subgroup are illustrated in Figure 1.

The demographic and clinical characteristics of patients with follicular lymphoma overall and by year of inclusion are reported in Supplementary Table S2. The overall population was aged on average 65.9 years, with no remarkable differences noticed over the years, and consistently, no linear trend of age categories was deduced over the years considered. Patients over 75 years old (30.2%) tend to be doubled compared to patients in the age range 71–75 (14.9%), and this ratio was observed in each year of inclusion. Follicular lymphoma was slightly more common in male subjects, with 54.1% of diagnosed case in the entire period, ranging from 51.7% in 2015 to 60.9% in 2020. The comorbidity profile evaluated by the CCI was not particularly severe. Table 1 describes the demographic and clinical features of the patient population divided according to the different treatment lines. Demographic variables were comparable across the lines of treatment, with mean age ranging 66.8 and 66 years and a slight predominance of male patients in all treated groups.

The patients in the age class of 18–70 years were around 56% at the start of the third or more line, those aged 71–75 years were 16–17%, and those in elderly class of ≥75 years were 26–27%. The CCI indicated a mild comorbidity profile, as it averaged between 1.1 and 1.3.

Table 2 depicts the pattern of patients with follicular lymphoma stratified by line of treatment and by calendar year of identification. The switchers to the subsequent line within a given year correspond to the incident patients of the subsequent line of the same year. A sustained switch rate within the same year was observed for all treatment lines and during all observation years. In detail, the proportion of patients who switched from 3L to 4L decreased from 27.9% in 2015 to 12.4% in 2019, those who switched from 4L to 5L from 31.8% in 2015 and 2016 to 19.2% in 2019, and those who switched from 5L onwards from 27% in 2015 to 17% in 2019.

The yearly mortality rates ranged from 5.6% (2015) to 10.3% (2018) for patients in 3L, from 2.7% (2015) to 9% (2019) for patients in 4L, and from 4.1% (2015) to 8.7% (2017) for patients in 5L.

**Figure 2:**
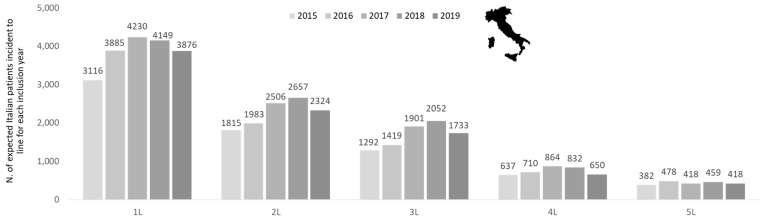
Estimated Italian patients incident to different treatment lines for follicular lymphoma by year of observation (2015–2019).

The data on patients incident to a specific line of treatment during each inclusion year were re-proportioned to the Italian population (Figure 2). Considering that, according to the census of the Italian Institute of Statistics, the national population consisted of approximately 60 million residents in the period 2015–2019 [25], the projections estimated a number of incident patients from 3116 (2015) to 4230 (2017) in 1L, from 1815 (2015) to 2657 (2018) in 2L, from 1292 (2015) to 2052 (2018) in 3L, from 637 (2015) to 864 (2017) in 4L, and from 382 (2015) to 478 (2016) in 5L (Figure 2).

**Figure 3:**
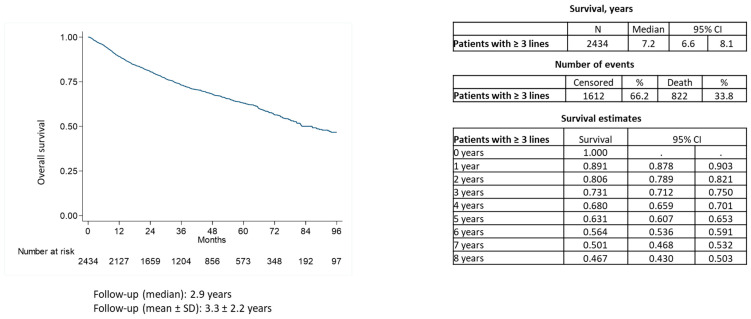
Kaplan Meier estimator of overall survival of patients with follicular lymphoma with ≥3 lines of treatment.

During a median follow-up of around 3 years, 34% of patients with ≥3L of therapy died. Median overall survival was of 7 years (Figure 3).

**Figure 4:**
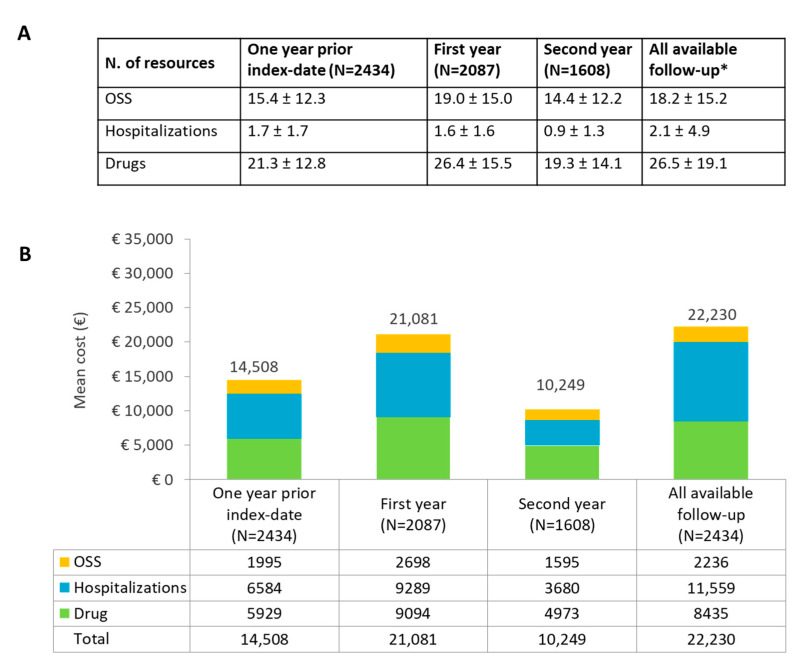
(**A**) Consumption of healthcare resources (mean ± SD) and (**B**) direct mean annual costs deriving from drugs, OSS (outpatient specialist services, namely diagnostic/laboratory tests and specialist visits) and hospital admissions. Evaluations were performed during the year before index date, during the first and second years, and for the whole available follow-up (* duration of all available follow-up: 3.3 ± 2.2 years).

Figure 4 illustrates the healthcare resources use per patient and the resulting mean direct annualized costs for the included follicular lymphoma population with ≥three treatment lines assessed in the year prior index date, at the first and second years of follow-up, and annualized considering the entire available follow-up that had a length of 3.3 ± 2.2 years. The analysis of healthcare use per patient revealed that most of the consumptions were related to drug prescriptions and delivery of OSS, and both items reached a zenith at the first year of follow-up (Figure 4A).

The total mean yearly expenses per patient for the management of follicular lymphoma with ≥three treatment lines were euro 14,508 in the year preceding the index date, euro 21,081 in the first year of follow-up, euro 10,249 in the second year of follow-up, and euro 22,230 during all available follow-up. Hospitalization and drugs were the most impactive cost items, representing each about 40–50% of overall healthcare expenditures, regardless of the time of evaluation (Figure 4B).

**Figure 5:**
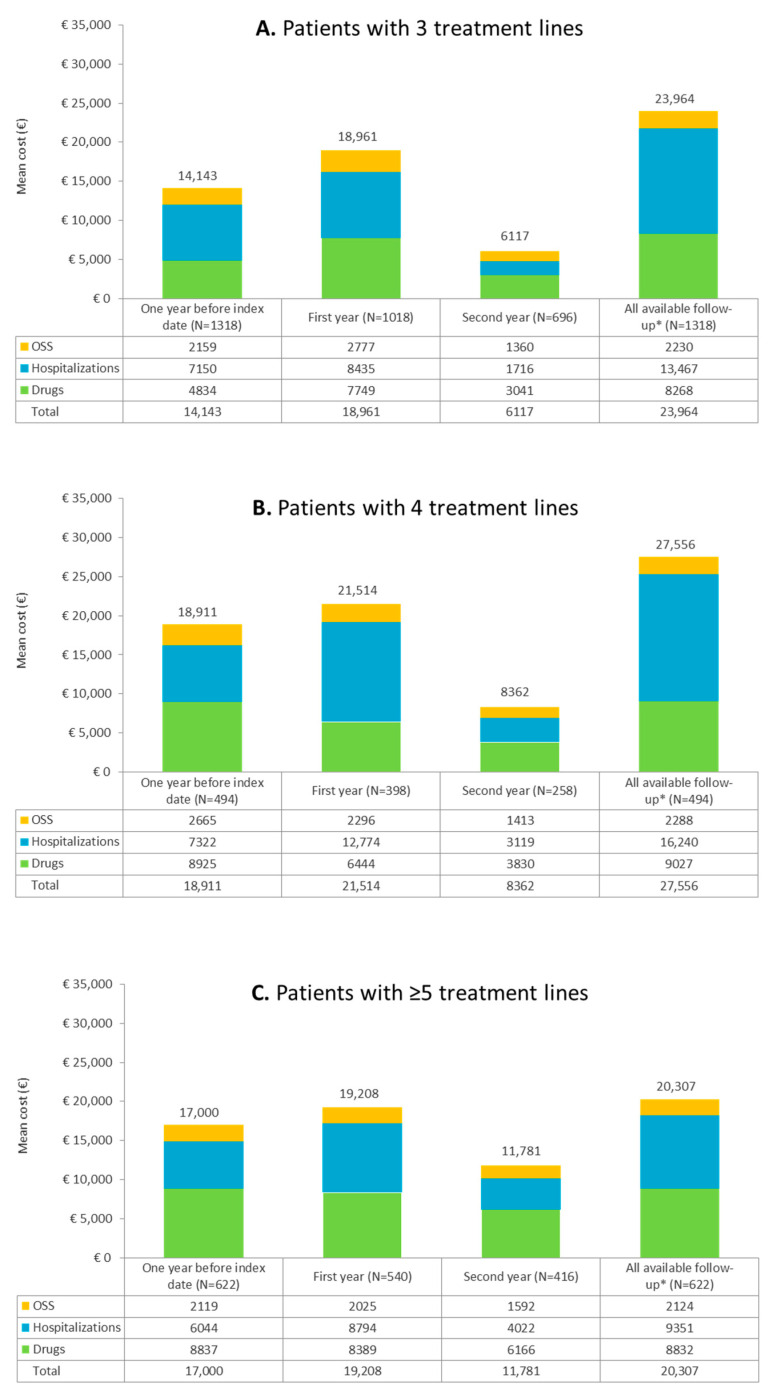
Direct mean annual costs during the year before index date, the first and second years of follow-up, and for the whole available follow-up for follicular lymphoma patients with (**A**) 3 treatment lines, (**B**) 4 treatment lines, and (**C**) ≥5 treatment lines. * Duration of all available follow-up: 2.6 ± 1.9 years for patients with 3L, 2.6 ± 1.8 years for patients with 4 L and 3.3 ± 2.1 years for patients with ≥5 treatment lines.

The cost analysis was then replicated at the same times mentioned above in the separate subgroups of follicular lymphoma patients with three, four, and five treatment lines (Figure 5A,B,C, respectively). For patients with 3L, the mean annual costs per patient were euro 14,143 in the year preceding the index date, euro 18,691 in the first year of follow-up, euro 6117 in the second year of follow-up, and euro 23,964 during all available follow-up, again mostly driven by drug expenses (Figure 5A). In the subgroup with 4L, the mean annual costs per patient were euro 18,911 in the year preceding the index date, euro 21,514 in the first year of follow-up, euro 8362 in the second year of follow-up, and euro 27,556 during all available follow-up, with drug costs as the most impactive item (Figure 5B). The patients with ≥5L had a mean annual cost per patient of euro 17,000 in the year preceding the index date, euro 19,208 in the first year of follow-up, euro 11,781 in the second year of follow-up, and euro 20,307 during all available follow-up, and once again mainly burdened by expenses for drugs (Figure 5C). Moreover, after this stratification by treatment lines, the economic analysis confirmed drug and hospitalization expenses as the weightiest cost items at all evaluation times.

To better characterize the clinical profile of the included population of follicular lymphoma patients with ≥three treatment lines, the frequencies (absolute number of patients and percentage) of the most commonly dispensed medications and most frequent hospitalization were assessed before and after the index date for the entire available follow-up (Table 3). Concerning drug prescriptions, antibacterials for systemic use, drugs for acid related disorders, antineoplastic agents, and systemic corticosteroids were widely prescribed to a growing extent during follow-up with respect to the time preceding the index date (Table 3A). About the hospitalizations, the largest proportion of the patients had admissions related to follicular lymphoma, which DRG falls into the Major Diagnostic Category (MDC) 17 myeloproliferative diseases and disorders (poorly differentiated neoplasms), specifically 68.5% prior the index date and 76.5% during the whole available follow-up. The second most common hospitalizations were due to the blood and blood forming organs and immunological disorders (MDC 16), namely 8.1% before the index date and 10.7% in the follow-up and those related to respiratory systems (5.3% before index date and 17.1% over follow-up). Other hospitalizations frequently detected were related to digestive/circulatory system and infections (Table 3B).

Among patients with follicular lymphoma under treatment, a total 366 patients had ASCT as ≥2 line of treatment, and among them, 79 (21.6%) underwent a subsequent treatment post-ASCT. The interval time from ASCT to next treatment was 17.3 ± 18.8 months, and the observational period after ASCT was 2.9 ± 2.1 years.

## 4. Discussion

Follicular lymphoma accounts for approximately 20% of all NHLs, with the highest incidence rates in the United States and Western Europe [26,27,28]. In the present analysis, 8406 patients with a diagnosis of follicular lymphoma were identified in a catchment area of approximately 13.3 million health-assisted individuals, which demographic characteristics as mean age around 65 years and the substantially comparable distribution between sexes were consistent with previous national and international data [3,4].

In our sample population, we found evidence of treatment in around 80% of patients, in line with an American real-world study by Batlevi et al. reporting 85% of patients with follicular lymphoma with at least a first line of treatment [29].

Although follicular lymphoma is still incurable, the landscape of life-prolonging drugs and therapeutic combinations is widening. Nevertheless, the heterogeneous clinical course of the disease results in variable therapeutic pathways, with several patients requiring multiple lines of treatment [21]. Consistently, among the patients included here, more than 40% of those who had undergone a therapy or procedure during the study period, had reached at least the third treatment line. Similar percentages were reported in a multicentre cohort study in which 441 patients out of 933 (47.3%) with follicular lymphoma had at least three lines of treatment [30].

A point that deserves much attention in this analysis is the constantly unneglectable proportion of switchers each year, regardless of treatment line and year of observation. This finding seems to highlight a still unmet clinical need, suggesting that many patients require a change in treatment, possibly suggestive of underlying disease progression [31,32,33]. This might be partly explicable by the natural course of follicular relapsing/refractory follicular lymphoma and the reduction of treatment options in later lines of therapy, that cause a significant burden for both patients and oncologists. In fact, while the first have to deal with an incurable disease and uncertain long-term prognosis, the clinicians are called to a complex decision-making process in an effort to find the proper balance between control of tumour progression, minimizing drug toxicity while also preserving patients’ quality of life [34].

Regarding the relapsing rate after ASCT, our data referred to a limited time span, however, they could be contextualized within a very recent multicentre Canadian study on 162 ASCT recipients for relapsed follicular lymphoma that reported over 12 years a time to progression of 57%, time to next treatment of 61%, progression-free survival of 51%. Thus, only about half of transplanted patients were able to achieve durable responses [35].

In the population analysed in the presented study, about one third of patients with ≥3L of therapy died over a median follow-up of 3 years, and the median overall survival was around 7 years. Our findings are in good agreement with the mentioned US real-world study by Batlevi and colleagues who reported a median overall survival of 8.75 years in patients after the third line of therapy, for an observation period of over 15 years, longer than that of the present study [29].

In this scenario, characterized by an increasingly complex management of patients with relapsing disease who need to proceed through several lines of treatment, the issue of resource allocation and cost sustainability acquires a relevant importance. In this real-world analysis, we evaluated the healthcare resource consumption per patient and the related direct annualized costs for the follicular lymphoma population with ≥three treatment lines assessed in the year prior index date, at the first, second year of follow-up, and for the entire available follow-up. Unsurprisingly, both consumptions and costs showed a peak at the first year of follow-up compared to the period before the index date (start of the third treatment line), with a declining trend in the second year; indeed, during the first year we observe consumption related to the start of the line of therapy, that could be followed by a period without treatment/procedures with consequently reduced cost. The weightiest cost items were hospitalizations and drugs that accounted for nearly half of the overall healthcare expenditures.

Over the past decade, several economic analyses have been conducted, but the majority of them were focused on the role of CD20 monoclonal antibodies (mainly rituximab) combined with CT used as first-line therapy or in subsequent treatment lines for relapsed/refractory disease [36,37,38]. Up to now, few studies have estimated this issue in the setting of follicular lymphoma patients with more than three lines of treatment. A real-world analysis on 598 insured US patients who started a treatment indicated for follicular lymphoma, confirmed the high economic burden associated with the disease, also highlighting the rise in healthcare consumptions and expenditures along with subsequent lines of therapy [31]. The authors reported that the most impactive categories for both all-cause and cancer-related resource utilization were office and other visits (expressed per patient per year, PPPY), as nearly all included patients underwent all-cause physician office visits and other outpatient visits during follow-up period, with increasing numbers from the first line (15.6 and 28.1 PPPY, respectively) to the fifth line of therapy (20.1 and 38.1 PPPY, respectively). Moreover, the costs were on the rise when progressing to subsequent lines of therapy, ranging from a mean annual cost per patient of USD 97,141 for first line to USD 424,758 for fifth-line therapy [31]. Our cost description must be interpreted taking into account the new therapeutic options available during the period analysed (idelalisib in 2015 and obizuntumab in 2019) as well as the guideline in force. The latter advised for later lines the use of chemoimmunotherapy (as rituximab with bendamustine) or immunotherapy alone, or idelalisib after a second line [8,39].

Poor data are currently available in Europe. A retrospective cohort study using population-based data from the French health insurance system analysed all subtypes of NHLs and indicated hospitalization and cancer-related drugs as the main cost drivers for disease management, in line with our findings [40]. The authors also emphasize how a proper and timely intervention in adverse events and concomitant diseases might have a beneficial impact on cost savings. The data from this analysis confirmed indeed that follicular lymphoma patients with ≥three treatment lines are also burdened by a complex clinical profile, as documented by the elevated use of non-cancer-related medications (like antibiotics, drugs for acid related disorders, or corticosteroids) and a relevant rate of hospitalizations from other causes than lymphoma itself; furthermore, the high rate of hospitalization related to respiratory system during follow-up could be related to the advent of COVID-19 pandemic.

This is one of the first real-time analyses on follicular lymphoma patients with ≥three treatment lines in Europe and to the best of our knowledge the first in Italy. Nevertheless, our results are subject to some limitations, primarily relating to the extrapolation of data from administrative flows. Such databases might lack some clinical information on individual patients such as disease severity, staging, and other clinical data that influence the therapeutic management of patients. For instance, rituximab is not always traceable in pharmaceutical databases for single patients, therefore, we included as under treatment also patients with a record for CT only, that could represent the proportion of patients for which it was not possible to link the rituximab use. The difficulty in the traceability of rituximab could have led also to an underestimation of the costs observed per patient. To this regard, the lack of detailed treatments for each patient could have affected the interpretation of cost description. Furthermore, by applying some gaps between consecutive CT records, there might be also a possible underestimation of refractory patients, who are, however, reported to be less than 10% of those treated with initial chemoimmunotherapy [15]. Nevertheless, such underestimation in the number of patients with later lines did not undermine the correct identification of the patients who received three or more lines analysed in the present manuscript. The degree of variation observed in the estimation of the number of patients with each line per year may depend on the methodology applied for the line identification. Administrative databases do not allow us to track the utilization of therapies within clinical trials, therefore some patients actually treated may be missing.

## 5. Conclusions

Our results further support the relevant clinical and economic burden associated with the management of follicular lymphoma in patients proceeding through several lines of treatment. The growing portfolio of novel drug classes to be combined with already established therapies has markedly improved life expectancy in relapsing/refractory disease, but the high mortality rates found in this analysis, as well as in previous data from other Western countries, indicate that further efforts are still necessary to optimize treatment pathway.

## Figures and Tables

**Table 1:** Demographic and clinical characteristics of patients with follicular lymphoma stratified by line of treatment. Continuous variables are presented as mean ± SD, and categorical variables as numbers and percentages in brackets.

Characteristics	≥3rd Line	3rd Line	4th Line	≥5th Line
N	2434	1318	494	622
Age, years	66.0 ± 13.5	65.9 ± 14.0	65.6 ± 13.4	66.8 ± 12.3
Age range				
18–70 years	1384 (56.9%)	741 (56.2%)	280 (56.7%)	351 (56.4%)
71–75 years	393 (16.1%)	215 (16.3%)	86 (17.4%)	109 (17.5%)
≥75 years	657 (27.0%)	362 (27.5%)	128 (25.9%)	162 (26.0%)
Male gender	1328 (54.6%)	722 (54.8%)	272 (55.1%)	334 (53.7%)
CCI	1.3 ± 1.8	1.3 ± 1.8	1.2 ± 17	1.1 ± 1.5

Abbreviations: CCI, Charlson comorbidity index.

**Table 2:** Number of patients with follicular lymphoma stratified by line of treatment and by calendar year of identification.

3L	2015	2016	2017	2018	2019
Prevalent patients that had a 3rd line up to the year	502	648	861	1057	1150
Incident to line	284 (56.6%)	312 (48.1%)	418 (48.5%)	451 (42.7%)	381 (33.1%)
Switch to subsequent line, same year	140 (27.9%)	156 (24.1%)	190 (22.1%)	183 (17.3%)	143 (12.4%)
Death, same year	28 (5.6%)	54 (8.3)	74 (8.6%)	109 (10.3%)	102 (8.9%)
Time (Years) from each calendar year to end of observation	4.8 ± 2.2	4.3 ± 2.0	3.9 ± 1.8	3.3 ± 1.8	2.9 ± 1.8
**4L**					
Prevalent patients that had a 4th line up to the year	264	330	403	470	480
Incident to line	140 (53)	156 (47.3)	190 (47.1)	183 (38.9)	143 (29.8)
Switch to subsequent line, same year	84 (31.8)	105 (31.8)	92 (22.8)	101 (21.5)	92 (19.2)
Death, same year	7 (2.7)	14 (4.2)	29 (7.2)	39 (8.3)	43 (9)
Time (Years) from each calendar year to end of observation	5.1 ± 1.9	4.5 ± 1.8	3.9 ± 1.7	3.4 ± 1.6	3.0 ± 1.7
**5L**					
Prevalent patients that had a 5th line up to the year	148	207	241	260	278
Incident to line	84 (56.8)	105 (50.7)	92 (38.2)	101 (38.8)	92 (33.1)
Switch to subsequent line, same year	40 (27)	51 (24.6)	67 (27.8)	57 (21.9)	47 (16.9)
Death, same year	6 (4.1)	10 (4.8)	21 (8.7)	21 (8.1)	22 (7.9)
Time (Years) from each calendar year to end of observation	4.6 ± 2.0	4.3 ± 1.7	3.9 ± 1.6	3.5 ± 1.6	2.9 ± 1.7

**Table 3:** Most frequent drugs (**A**) and hospitalization (**B**) in follicular lymphoma patients with ≥3 treatment lines before and after the index date (all available follow-up). Data are given as number of patients and percentage in brackets.

**(** **A). Most Frequent Drugs**			
**ATC**	**Description**	**Before Index Date (N = 2434)**	**All Available Follow-Up (N = 2434)**
J01	Antibacterials for systemic use	2155 (88.5%)	2286 (93.9%)
A02	Drugs for acid related disorders	2081 (85.5%)	2154 (88.5%)
L01	Antineoplastic agents	1902 (78.1%)	1915 (78.7%)
H02	Corticosteroids for systemic use	1728 (71.0%)	1954 (80.3%)
B01	Antithrombotic agents	1294 (53.2%)	1555 (63.9%)
M04	Antigout preparations	1215 (49.9%)	1159 (47.6%)
J05	Antivirals for systemic use	1079 (44.3%)	1478 (60.7%)
**(B). Most Frequent Hospitalizations**			
**MDC**	**Description**	**Before index date (N = 2434)**	**All available follow-up (N = 2434)**
17	Myeloproliferative DDs (poorly differentiated neoplasms) *	1668 (68.5%)	1861 (76.5%)
16	Blood and blood forming organs and immunological disorders	196 (8.1%)	261 (10.7%)
4	Respiratory system	129 (5.3%)	415 (17.1%)
23	Factors influencing health status	109 (4.5%)	160 (6.6%)
6	Digestive system	96 (3.9%)	185 (7.6%)
5	Circulatory system	76 (3.1%)	231 (9.5%)
18	Infectious and parasitic DDs	65 (2.7%)	283 (11.6%)

Abbreviations: ATC, Anatomical Therapeutic Chemical; DDs, diseases and disorders; MDC, Major Diagnostic Category. * Follicular lymphoma is included in MDC 17.

## Data Availability

All data used for the current study are available upon reasonable request next to CliCon s.r.l. which is the body entitled of data treatment and analysis by Local Health Units.

## Supplementary Materials

The following supporting information can be downloaded at: https://www.mdpi.com/article/10.3390/cancers15174403/s1, Table S1. Treatments and procedures with the related codes used in patients with follicular lymphoma; Table S2. Demographic and clinical characteristics of patients with follicular lymphoma overall and by year of inclusion. Continuous variables are presented as mean ± SD, and categorical variables as numbers and percentages in brackets.
