# Supplementary material for: Burden of Illness in Follicular Lymphoma with Multiple Lines of Treatment, Italian RWE Analysis
Source: Cancers (Basel). 2023 Sep 2;15(17):4403. doi: 10.3390/cancers15174403 (PMC10486445; doi:10.3390/cancers15174403)
Supplement: Supplementary file 1 [file cancers-15-04403-s001.zip › cancers-2504511-supplementary.pdf]

Burden of illness in follicular lymphoma with multiple lines of treatment, Italian RWE analysis

Supplementary materials

Table S1. Treatments and procedures with the related codes used in patients with follicular lymphoma.

Treatment	Identification codes
Rituximab ± aspecific CT	ATC codes: L01XC02/L01FA01 **OR** minsan G00463001 ± aspecific CT (DRG code: 410 **OR** hospital/ambulatory procedures 99.25, 99.28, **OR** ATC L01 excluded the treatments listed below).
Aspecific CT	DRG code: 410 **OR** hospital/ambulatory procedures 99.25, 99.28, **OR** ATC L01 excluded the treatments listed below
Obinutuzumab	ATC code: L01XC15/L01FA03
Idelalisib	ATC code: L01EM01
Ibritumomab tiuxetan	ATC code: V10XX02
Lenalidomide	ATC code: L04XA04
ASCT	ICD-9-CM code (procedures): 41.04 autologous hematopoietic stem cell transplant without purging; 41.07 autologous hematopoietic stem cell transplant with purging

Abbreviations: ATC, Anatomical Therapeutic Chemical (ATC); ASCT, autologous stem cell transplantation; CT, chemotherapy; DRG, Diagnosis Related Group; ICD-9-CM, International Classification of Diseases, Ninth Revision, Clinical Modification.

Table S2 Demographic and clinical characteristics of patients with follicular lymphoma overall and by year of inclusion. Continuous variables are presented as mean ± SD, and categorical variables as numbers and percentages in brackets.

Characteristics	Overall	2015	2016	2017	2018	2019
N	7,021	1270	1263	1389	1439	1453
Age, years	65.9 ± 15.3	65.3 ± 14.5	65.7 ± 15.2	65.7 ± 15.2	65.8 ± 16.2	67.0 ± 15.4
Age range						
18-70 years	3,855 (54.9%)	750 (59.1%)	702 (55.6%)	783 (56.4%)	773 (53.7%)	733 (50.4%)
71-75 years	1,043 (14.9%)	188 (14.8%)	192 (15.2%)	199 (14.3%)	202 (14.0%)	228 (15.7%)
≥75 years	2,123 (30.2%)	332 (26.1%)	369 (29.2%)	407 (29.3%)	464 (32.2%)	492 (33.9%)
Male gender	3,796 (54.1%)	657 (51.7%)	710 (56.2%)	764 (55.0%)	765 (53.2%)	774 (53.3%)
CCI	1.3 ± 1.7	1.4 ± 1.8	1.4 ± 1.8	1.3 ± 1.7	1.3 ± 1.7	1.2 ± 1.6

Abbreviations: CCI, Charlson comorbidity index.
